# The Use of Fourier Transform Infrared Microspectroscopy for the Determination of Biochemical Anomalies of the Hippocampal Formation Characteristic for the Kindling Model of Seizures

**DOI:** 10.1021/acschemneuro.1c00642

**Published:** 2021-11-24

**Authors:** Marzena M. Rugiel, Zuzanna K. Setkowicz, Agnieszka K. Drozdz, Krzysztof J. Janeczko, Justyna Kutorasińska, Joanna G. Chwiej

**Affiliations:** † Faculty of Physics and Applied Computer Science, AGH University of Science and Technology, A. Mickiewicza 30, Krakow 30-059, Poland; ‡ Institute of Zoology and Biomedical Research, Jagiellonian University, Golebia 24, Krakow 31-007, Poland; § Maria Curie-Sklodowska University, Institute of Biological Sciences, Akademicka 19, Lublin 20-033, Poland

**Keywords:** rat kindling model of epilepsy, transauricural electroshocks, topographic and quantitative biochemical analysis, Fourier transform infrared microspectroscopy, clonic and tonic seizures, biochemical anomalies

## Abstract

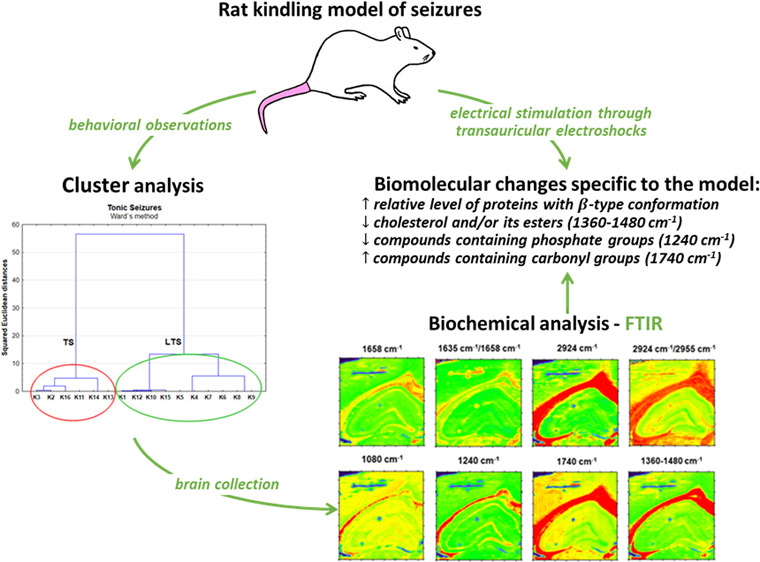

The animal models of seizures and/or epilepsy are widely used to identify the pathomechanisms of the disease as well as to look for and test the new antiseizure therapies. The understanding of the mechanisms of action of new drugs and evaluation of their safety in animals require previous knowledge concerning the biomolecular anomalies characteristic for the particular model. Among different models of seizures, one of the most widely used is the kindling model that was also applied in our study. To examine the influence of multiple transauricular electroshocks on the biochemical composition of rat hippocampal formation, Fourier transform infrared (FT-IR) microspectrosopy was utilized. The chemical mapping of the main absorption bands and their ratios allowed us to detect significant anomalies in both the distribution and structure of main biomolecules for electrically stimulated rats. They included an increased relative content of proteins with β-sheet conformation (an increased ratio of the absorbance at the wavenumbers of 1635 and 1658 cm^–1^), a decreased level of cholesterol and/or its esters and compounds containing phosphate groups (a diminished intensity of the massif of 1360–1480 cm^–1^ and the band at 1240 cm^–1^), as well as increased accumulation of carbohydrates and the compounds containing carbonyl groups (increased intensity of the bands at 1080 and 1740 cm^–1^, respectively). The observed biomolecular abnormalities seem to be the consequence of lipid peroxidation promoted by reactive oxygen species as well as the mobilization of glucose that resulted from the increased demand to energy during postelectroshock seizures.

## Introduction

Animal models of seizures are used both to develop new anticonvulsants and to observe processes associated with epileptogenesis.^1,2^ An optimal experimental model of epileptic seizures should provide a reliable way of inducing a stable, epileptic-like condition as well as predictability of response to treatment.^2,3^ Among different animal models of seizures, *post status epilepticus* (SE) models and kindling models should be mainly distinguished. In the first case, SE is induced by the administration of substances with proconvulsive properties, such as kainic acid, pilocarpine, or lithium+pilocarpine.^1,3^ A characteristic feature of *post SE* models is amygdala damage and hippocampal sclerosis, including cytoarchitectonic disorganization and death of pyramidal neurons.^1,4−6^ Numerous studies have indicated that these changes resemble anomalies occurring in the temporal lobe areas of patients suffering from temporal lobe epilepsy (TLE).^3,5,7−10^

Kindling models are based on chronic exposure to a subliminal stimulus (chemical or electrical) of proconvulsive nature.^3^ As a result of 1–3 weeks of electrical stimulation of the limbic system structures or repeated administration of the proconvulsive agent, the seizure threshold is reduced and seizures with a gradually increasing severity and duration appear.^1,3^ The occurrence of spontaneous convulsions in this model is a rare phenomenon; therefore, stimulus-induced seizures are usually the subject of research. The nature of recorded seizures as well as their sensitivity to pharmaceuticals are very similar in *post SE* and kindling models.^1,3,11^ However, in the latter, neuropathological changes typical for TLE such as hippocampal sclerosis are not noted.^12,13^ On the other hand, in the brains of rats subjected to repeated stimulation, as observed in *post SE* models, the loss of interneurons inside the dentate gyrus is observed.^12,14^

Our previous research has focused mainly on the pilocarpine model of seizures. To study the mechanisms connected with the pathogenesis and progress of epilepsy in the model, we successfully used advanced methods of atomic (X-ray fluorescence microscopy) and molecular [Fourier transform infrared (FT-IR) and Raman microspectroscopy] spectroscopy.^15−20^ The results obtained using them indicate that the process of excitotoxicity, mossy fiber sprouting, iron-catalyzed oxidative stress, and decreased creatine kinase activity may underlay the neurodegenerative changes in the hippocampus and spontaneous seizure activity in the chronic phase of the pilocarpine model of epilepsy.

Our preliminary study carried out on rats subjected to repetitive electrical stimulation showed that the potential mechanism responsible for the occurrence of the kindling phenomenon and pathological changes within the hippocampus in this model of seizures is the mossy fiber sprouting but not the excitotoxic injury.^21^ The mentioned results were based on the elemental analysis of brain slices using synchrotron X-ray fluorescence microscopy. In turn, in the present study, we used FT-IR microspectroscopy to determine the changes in the content and structure of main biological macromolecules that occur in the hippocampal formation of rats subjected to transauricural electroshocks, which lead to the appearance of the kindling phenomenon in animals. The obtained biochemical data were analyzed with respect to the behavioral parameters describing the duration and intensity of seizures occurring in the answer to stimulation. A very useful tool for this purpose turned out to be the cluster analysis, allowing for grouping of animals based on the similarities in their behavior after multiple use of stimuli.

## Results and Discussion

### Analysis of the Influence of Electrical Stimulation on the Biochemical Composition of Hippocampal Formation

To verify the hypothesis that transauricular electrical stimulation modifies the content and structure of biomolecules within the examined cellular layers of hippocampal formation, the biochemical composition of this brain area in rats subjected to multiple stimulation (K group) and controls (N group) was compared. The carried comparisons included the topographic analysis of chemical maps, presenting the distribution of the absorption bands characteristic for the main biomolecules, the quantitative determination of biochemical parameter values for particular animals and experimental groups, as well as statistical evaluation of the differences between the examined animal populations (*U* Mann–Whitney test).

#### Distribution and Structural Changes of Proteins

**Figure 1:**
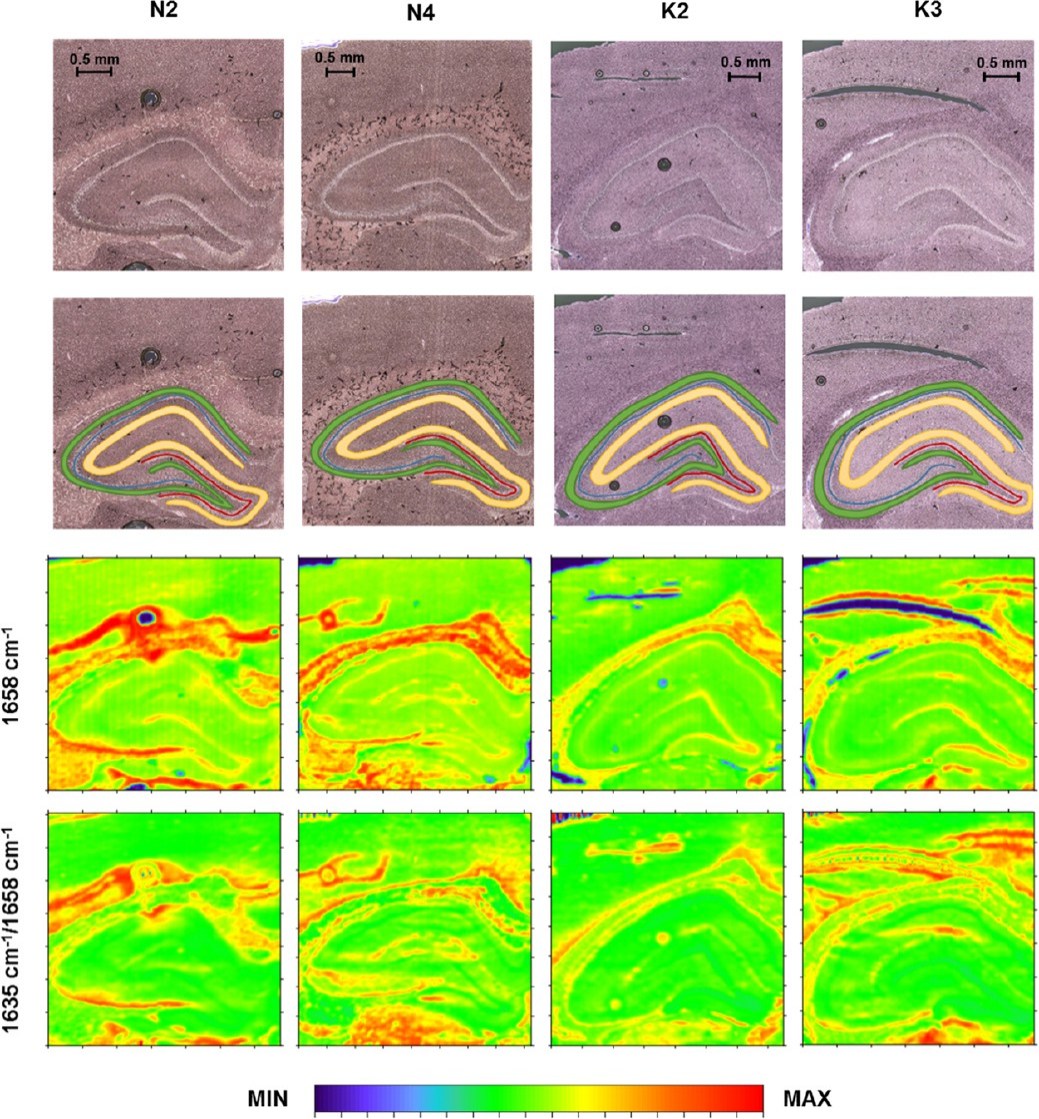
Example chemical maps of the amide I band and the ratio of absorbance at the wavenumbers of 1635 and 1658 cm^–1^ obtained for the hippocampal formation taken from rats exposed to electrical stimulation (K) and from the control animals (N) together with the microscopic views of the scanned tissue areas (first row) and the localization of pixels (second row) taken for quantitative analysis in the case of granular (red), pyramidal (blue), multiform (green), and molecular (yellow) layers.

**Figure 2:**
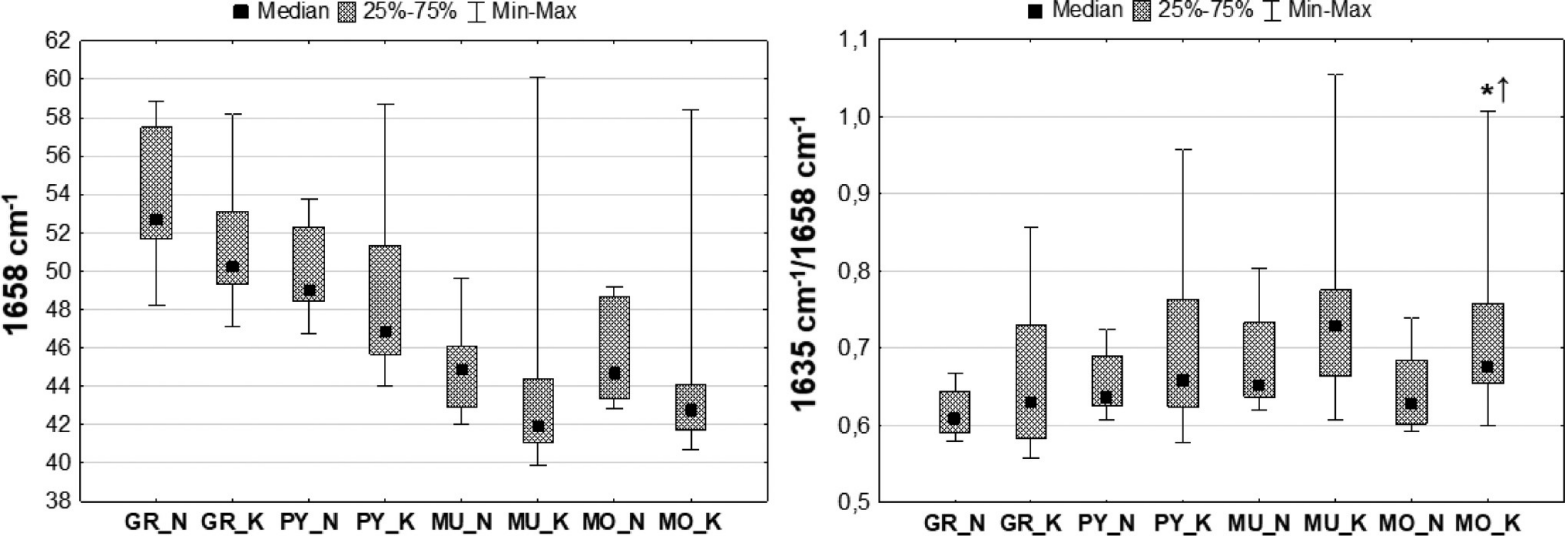
Box-and-whisker plots presenting the spread of the values of the amide I band intensity and the ratio of absorbance at 1635 and 1638 cm^–1^ in the examined cellular layers (GR-granular, PY-pyramidal, MU-multiform, and MO-molecular) for the K and N groups. Median, interquartile span, and minimal-maximal values are marked as a little square, a box, and whiskers, respectively. Statistically relevant increase (Mann–Whitney *U* test, 95% confidence level) in the ratio of absorbance at 1635 and 1638 cm^–1^ for the K group was marked as *↑.

The analysis of chemical maps presenting the amide I band intensity did not show obvious changes in protein accumulation within the hippocampal formation of the electrically stimulated rats (Figure 1). Such a result was also confirmed by further quantitative evaluation, followed by the Mann–Whitney *U* test. Although the level of proteins did not change in animals subjected to electrical stimulation, they presented an elevated ratio of absorbance at the wavenumbers of 1635 and 1658 cm^–1^ in the molecular layer, which may suggest an increase in the relative content of proteins with the β-type secondary structure within this hippocampal area. Although the statistical relevance of differences was found only for the molecular layer, a similar trend was observed for all examined cells, as one can see in Figure 2, presenting the spread of the ratio of absorbance at the wavenumbers of 1635 and 1658 cm^–1^ within the K and N groups, respectively.

#### Distribution and Structural Changes of Lipids

**Figure 3:**
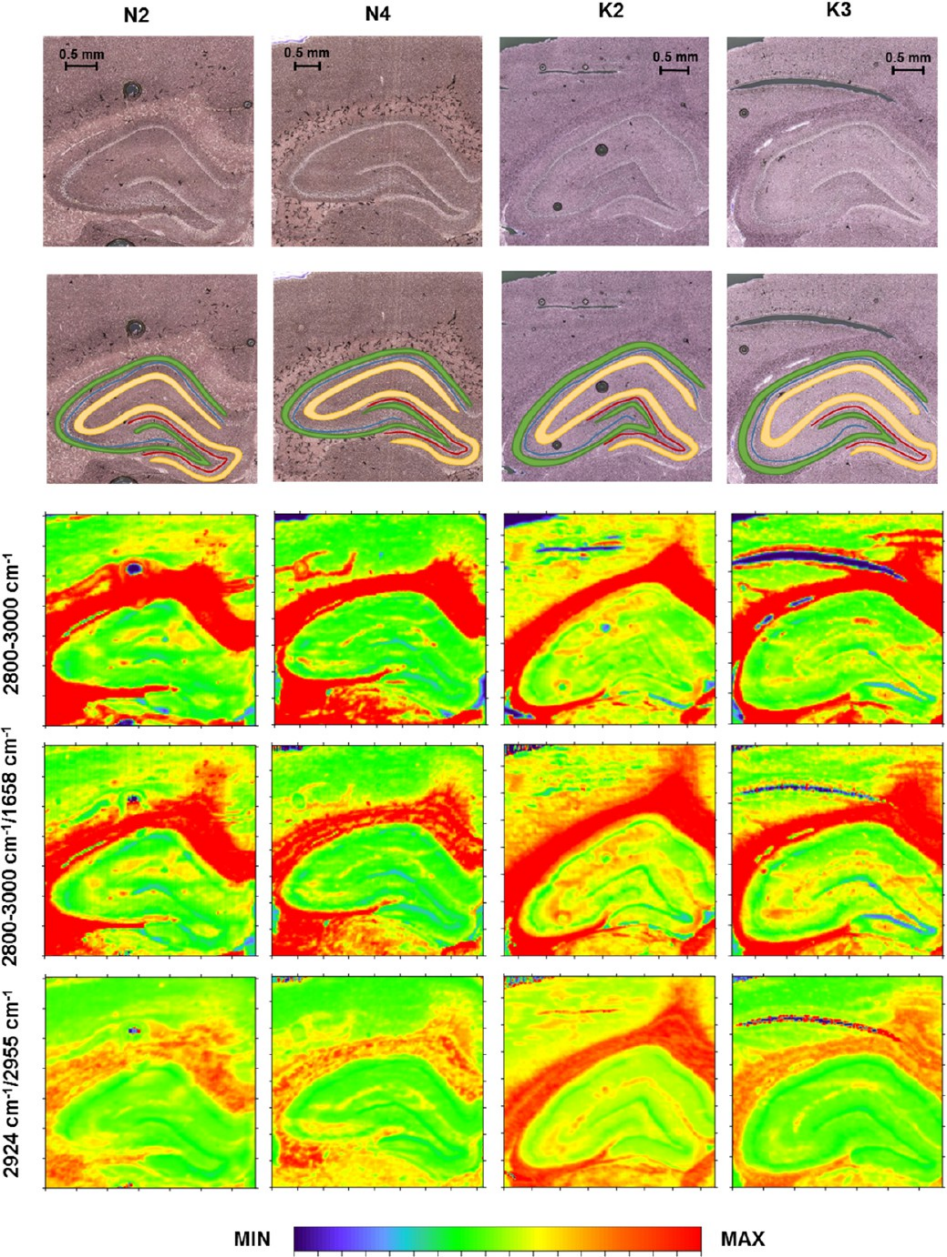
Example chemical maps of lipid massif 2800–3000 cm^–1^ and of the intensity ratios of the selected bands (2800–3000/1658 and 2924/2955 cm^–1^) obtained for the hippocampal formation taken from rats subjected to electrical stimulation (K) and from the control animals (N) together with the microscopic views of the scanned tissue areas (first row) and the localization of the pixels (second row) taken for quantitative analysis in the case of granular (red), pyramidal (blue), multiform (green), and molecular (yellow) layers.

**Figure 4:**
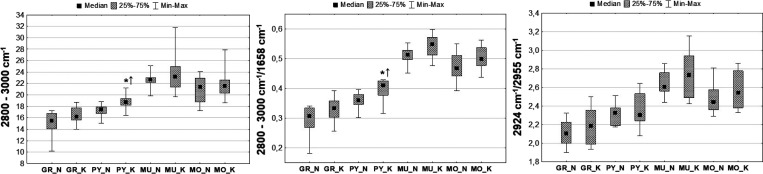
Box-and-whisker plots presenting the spread of the 2800–3000 cm^–1^ massif intensity, the ratio of lipid massif and amide I band intensities, as well as the ratio of the bands at 2924 and 2955 cm^–1^ in the examined hippocampal layers (GR-granular, PY-pyramidal, MU-multiform, and MO-molecular) for the K and N groups. Median, interquartile span, and minimal-maximal values are marked as a little square, a box, and whiskers, respectively. Statistically significant differences (Mann–Whitney *U* test, 95% confidence level) between animals subjected to electroshocks (K) and naive controls (N) are marked with *. Statistically relevant increases found for the K group are marked as ↑.

The Mann–Whitney *U* test did not show any statistically significant differences in the ratio of 2924 and 2955 cm^–1^ lipid band intensities between the groups N and K (Figure 3). In turn, as one can see from Figure 4, the absolute accumulation of lipids (measured by the intensity of massif 2800–3000 cm^–1^) and the relative intensity of lipid massif compared to the amide I band (2800–3000 cm^–1^/1658 cm^–1^) was elevated within the pyramidal layer of animals from the K group.

#### Accumulation of Compounds Containing Phosphate Groups

**Figure 5:**
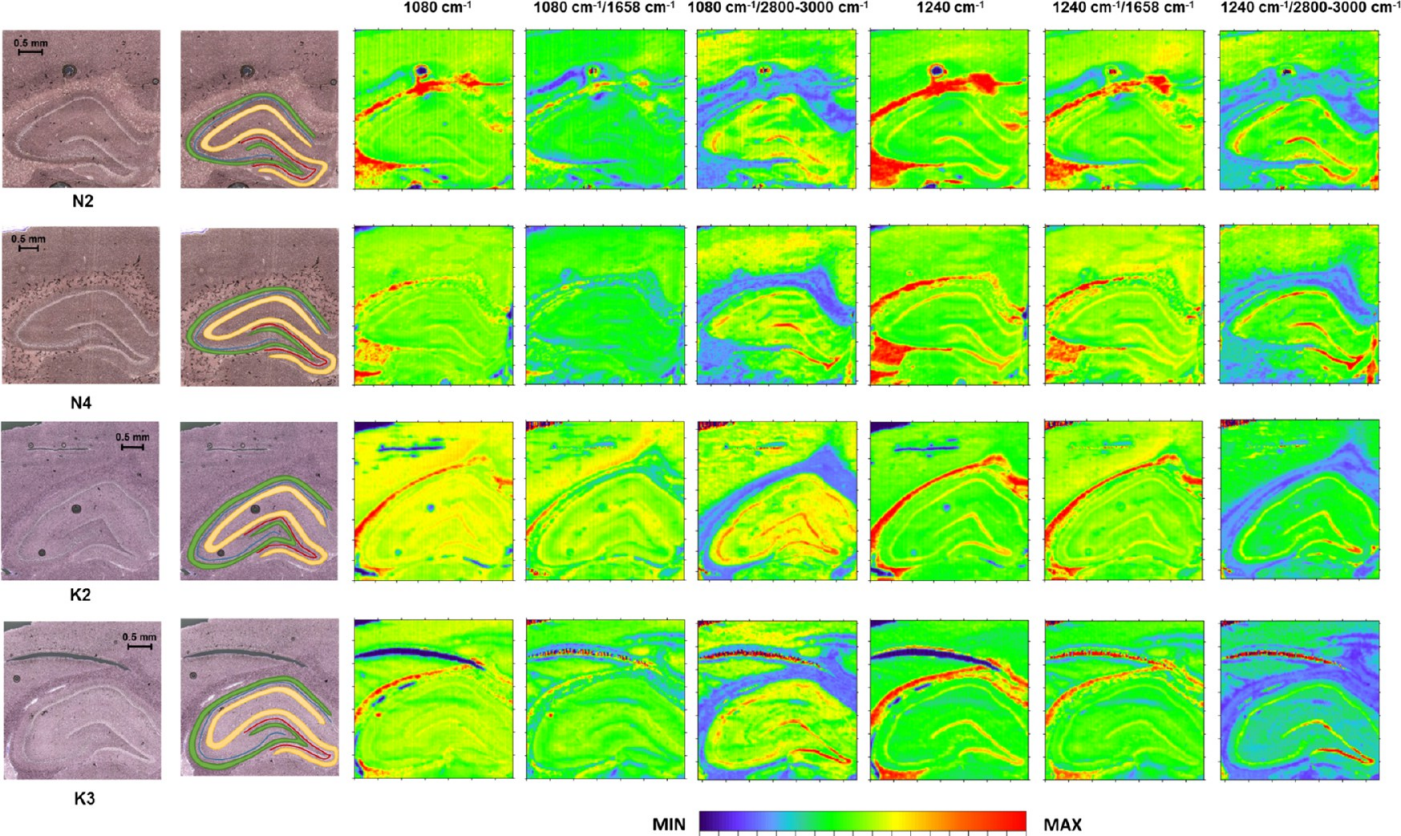
Example chemical maps of the absorption bands at 1080 and 1240 cm^–1^ as well as of their relative intensities, which were obtained for the hippocampal formation taken from rats exposed to electrical stimulation (K) and control animals (N) together with the microscopic views of the scanned tissue areas (first column) and the localization of the pixels (second column) taken for quantitative analysis in the case of granular (red), pyramidal (blue), multiform (green), and molecular (yellow) layers.

**Figure 6:**
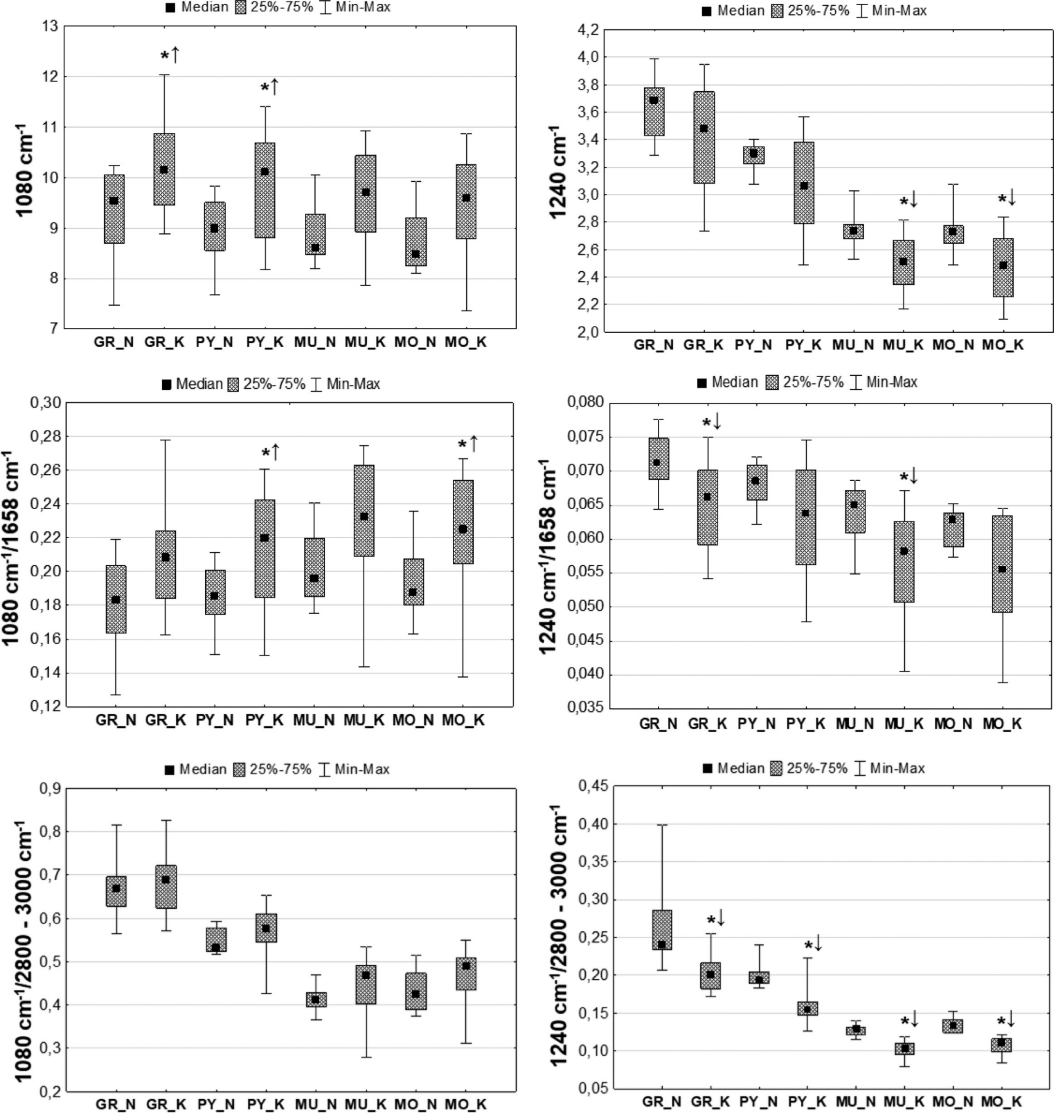
Box-and-whisker plots presenting the spread of the values of the 1080 and 1240 cm^–1^ band intensities and their relative (with respect to proteins and lipids) contents within the examined cellular layers (GR-granular, PY-pyramidal, MU-multiform, and MO-molecular) for the K and N groups. Median, interquartile span, and minimal-maximal values are marked as a little square, a box, and whiskers, respectively. Statistically significant differences (Mann–Whitney *U* test, 95% confidence level) between animals subjected to electroshocks (K) and naive controls (N) are marked with *. Statistically relevant increases found for the K group are marked as ↑, while the decreases as ↓.

As one can see from Figures 5 and 6, a statistically relevant (*p* < 0.05) increase of 1080 cm^–1^ band intensity was found in the granular and pyramidal layers of hippocampal formation for animals representing the K group, which could suggest the elevation of the level of compounds containing the phosphate groups in the answer to the electroshocks. The observed anomalies were not, however, positively correlated with the intensity of the band of 1240 cm^–1^, which decreased in the multiform and molecular layer for the electrically stimulated animals. Also, the relative levels of these two absorption bands were not positively correlated. When the relative intensity of the 1080 cm^–1^ absorption band with respect to proteins was elevated within the pyramidal and molecular layers, the ratio of the absorption bands at 1240 and 1658 cm^–1^ was significantly lower in the granular and multiform cellular layers. What is more, for all four layers, the intensity of the 1240 cm^–1^ band with respect to the massif 2800–3000 cm^–1^ decreased.

#### Distribution of Compounds Containing Carbonyl Groups

**Figure 7:**
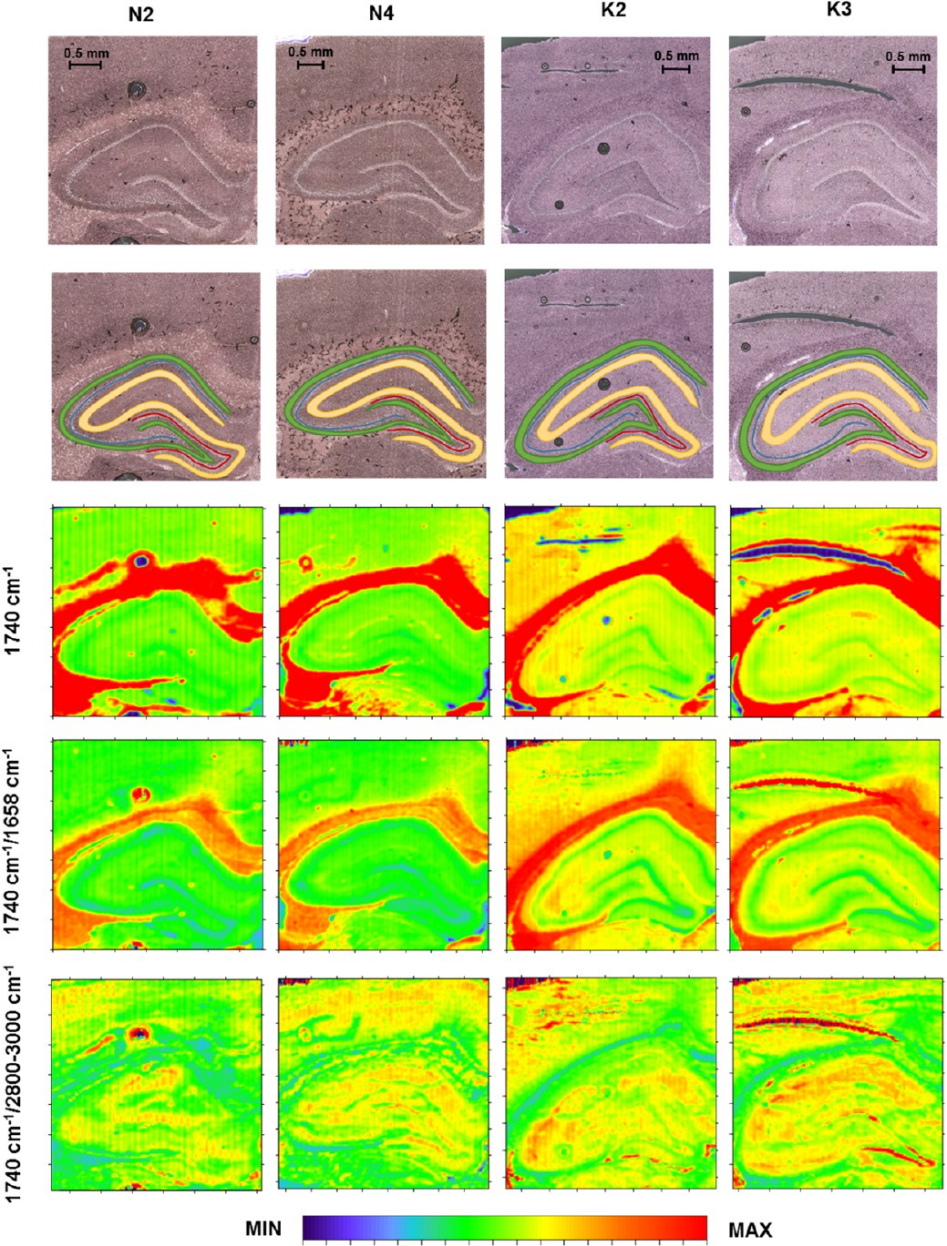
Example chemical maps presenting the absolute and the relative (comparing to the amide I band and lipid massif) intensities of the 1740 cm^–1^ absorption band, which were obtained for the hippocampal formation taken from the rats exposed to electrical stimulation (K) and from the control animals (N). The first row shows the microscopic views of the scanned tissue areas, while the second row shows the localization of the pixels (second row) taken for quantitative analysis in the case of granular (red), pyramidal (blue), multiform (green), and molecular (yellow) layers.

**Figure 8:**
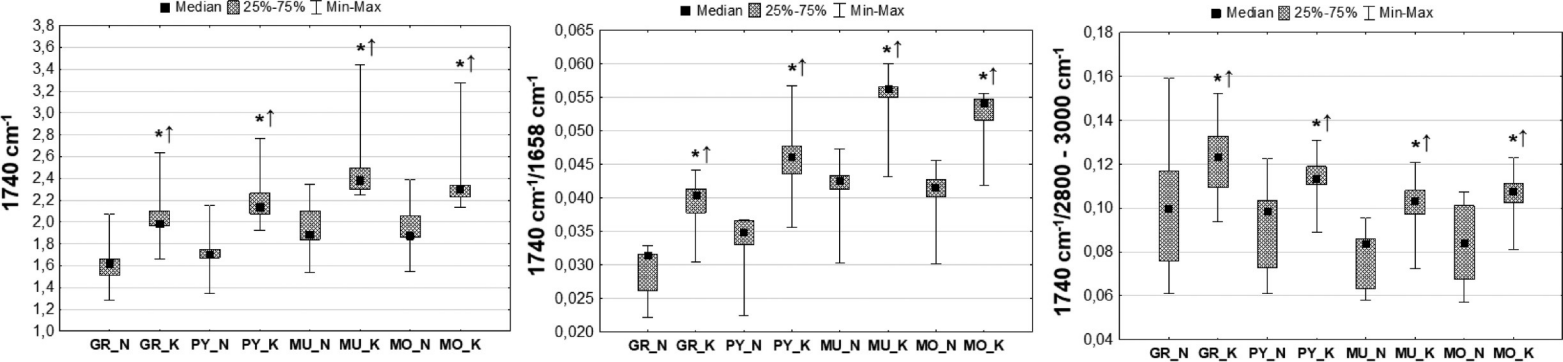
Box-and-whisker plots presenting the spread of the absolute and relative (with respect to proteins and lipids) intensity of the 1740 cm^–1^ band within the examined cellular layers (GR-granular, PY-pyramidal, MU-multiform, and MO-molecular) for the K and N groups. Median, interquartile span, and minimal-maximal values are marked as a little square, a box, and whiskers, respectively. Statistically significant differences (Mann–Whitney *U* test, 95% confidence level) between animals subjected to electroshocks (K) and naive controls (N) are marked with *. Statistically relevant increases found for the K group are marked as ↑.

For all examined hippocampal layers, the intensity of the 1740 cm^–1^ absorption band was significantly elevated in animals exposed to electrical stimulation. Also, the relative contents of this IR band (1740 cm^–1^/1658 cm^–1^ and 1740 cm^–1^/2800–3000 cm^–1^) were higher in rats subjected to electroshocks compared to controls, as shown in Figure 7, presenting chemical maps obtained for selected animals representing K and N groups and in Figure 8, where the spread of the analyzed biochemical parameters is presented as box-and-whisker plots.

#### Accumulation of Compounds Containing Methyl and Methylene Groups (Lipids, Cholesterol, and/or Cholesterol Esters)

**Figure 9:**
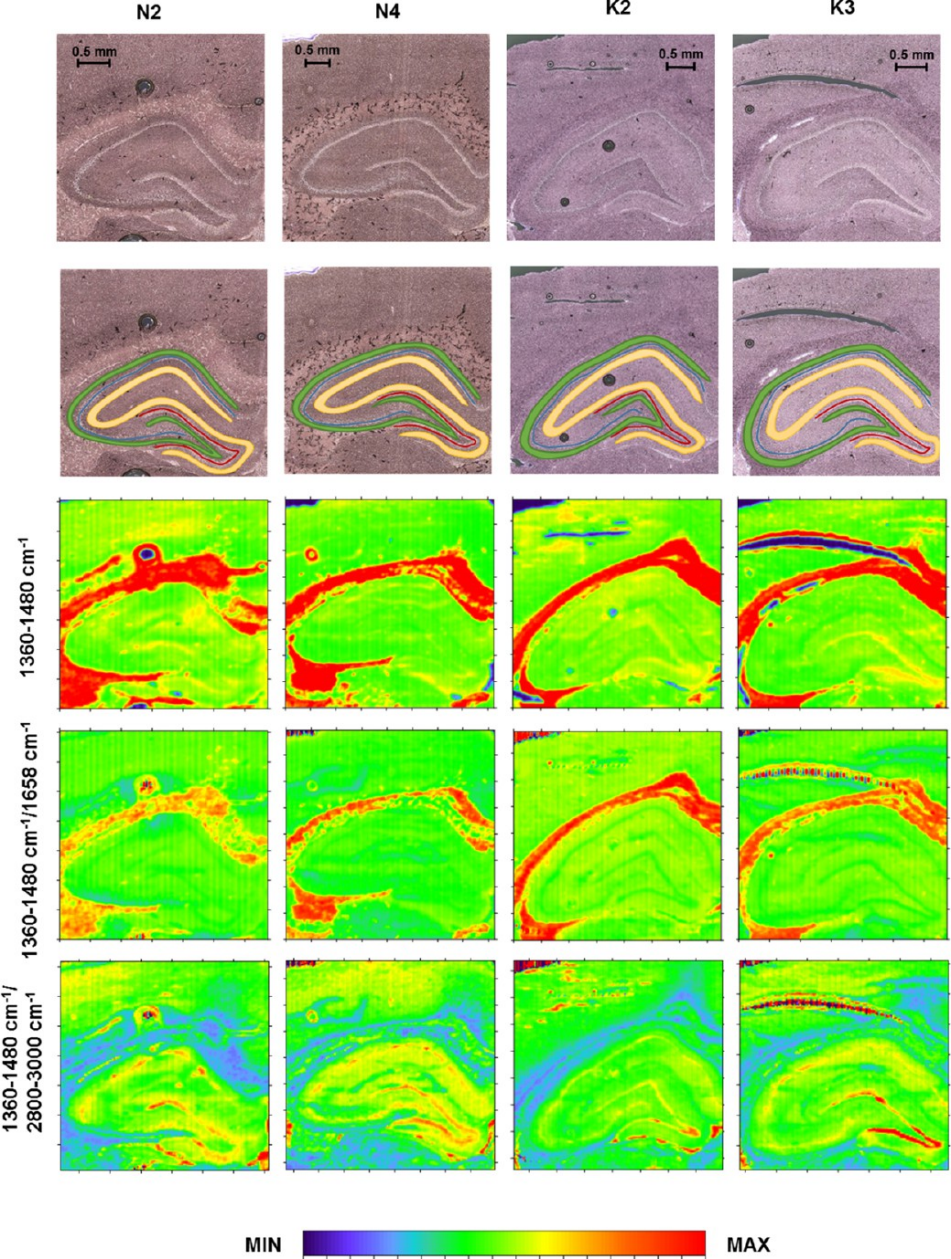
Example chemical maps presenting the absolute and the relative (compared to the amide I band and lipid massif) intensities of the massif of 1360–1480 cm^–1^, which were obtained for the hippocampal formation taken from the rats exposed to electrical stimulation (K) and from the control animals (N). The first row shows microscopic views of the scanned tissue areas, while the second shows the localization of the pixels (second row) taken for quantitative analysis in the case of granular (red), pyramidal (blue), multiform (green), and molecular (yellow) layers.

**Figure 10:**
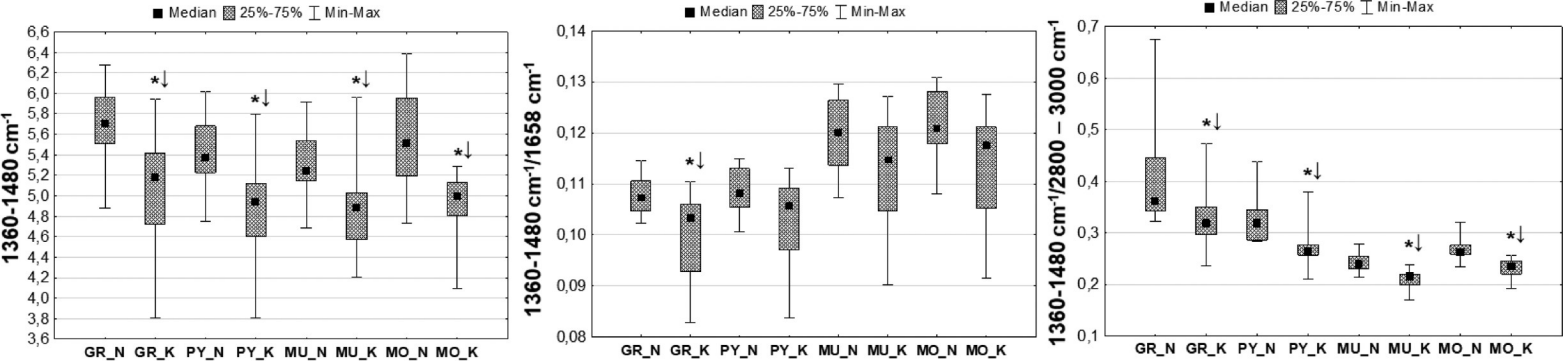
Box-and-whisker plots presenting the spread of the absolute and the relative (in respect to proteins and lipids) intensity of the massif of 1360–1480 cm^–1^ within the examined cellular layers (GR-granular, PY-pyramidal, MU-multiform, and MO-molecular) for the K and N groups. Median, interquartile span, and minimal-maximal values are marked as a little square, a box, and whiskers, respectively. Statistically significant differences (Mann–Whitney *U* test, 95% confidence level) between animals subjected to electroshocks (K) and naive controls (N) are marked with *. Statistically relevant decreases found for the K group are marked as ↓.

The massif occurring at the wavenumber range of 1360–1480 cm^–1^ is very complex, and its presence in the IR spectrum of the brain is connected with different bending vibrations of methyl and methylene groups originating from lipids, cholesterol, and/or cholesterol esters. The statistically relevant (*p* < 0.05) decrease in absorption at this wavenumber region for all the examined cellular layers was observed in animals exposed to electroshocks. A similar relation was found for the intensity ratio of the massifs of 1360–1480 cm^–1^ and 2800–3000 cm^–1^. The intensity of the massif in comparison to proteins (1360–1480 cm^–1^/1658 cm^–1^) was generally lower in the K group, but the observed decrease was statistically relevant only within the granular layer. These relations were presented on chemical maps and box-and-whiskers charts in Figures 9 and 10, respectively.

### Analysis of the Relationships between the Biochemical Anomalies and Parameters Describing Electroshock-Induced Changes in Animal Behavior

**Figure 11:**
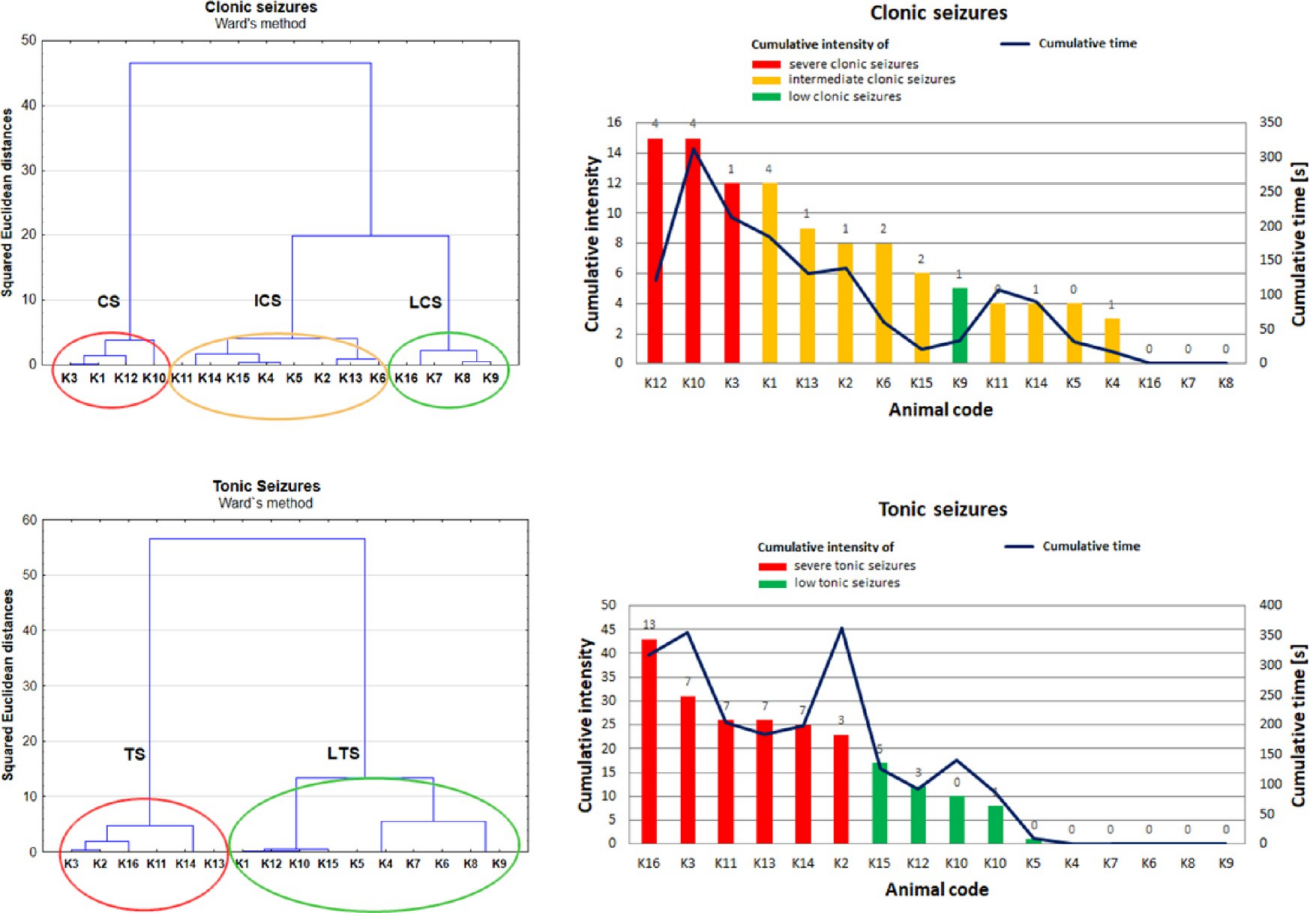
Comparison of dendrograms that were obtained based on the cumulative (totaled over the 21 day stimulation period) intensity and durations of the clonic and tonic seizures (independently). Ward’s method of clustering and the squared Euclidean distance as a measure of similarity between observations were used. Red, yellow, and green bars show, respectively, the groups of animals with severe, intermediate, and low seizures, which were extracted based on the cluster analysis. The values above the bars indicate the number of seizures of maximal intensity occurring during the 21 stimulation days.

During the 21 day long experiment, the animal behavior was monitored for 1 h from stimulation, and the information concerning the intensity and duration of tonic and clonic seizures was recorded daily. As one can notice from Figure 11 showing the cumulative (for the whole experiment) values of the measured behavioral parameters, the rats presented quite a large variability in their susceptibility to electroshocks. Therefore, in the next step of the study, the animals were divided into subgroups, and the cluster analysis was applied to achieve this goal. This method of multivariate analysis is a useful statistical tool to search for patterns in a collection of objects by grouping them into clusters. It is realized in such a way that the observations within each cluster have a maximal degree of similarity and a minimal degree of association to observations outside this cluster. The obtained results are shown in the form of a graph called a dendrogram.^22^

In the present study, a nonsupervised classification of electrically stimulated animals was carried out using Ward’s method with a squared Euclidean distance as a measure of similarity between cases. The hierarchical Ward’s method of clustering uses the analysis of variance approach to create connections between observations, minimizing the sum of deviation squares within the two clusters that can be formed at each step of analysis.^22^ In Figure 11, one can compare the results of cluster analysis done, independently, based on the behavioral parameters describing the tonic and clonic seizures.

As one can see in Figure 11, the shape of the dendrograms obtained based on the parameters describing clonic and tonic seizures presents significant dissimilarities. For clonic seizures, three distinct clusters, severe clonic seizures (CS), intermediate clonic seizures (ICS), and light clonic seizures (LCS), were distinguished. Cluster CS included animals in which electrical stimulation induced severe responses manifesting by the high intensity and duration of clonic seizures. The animals from cluster ICS presented seizures of intermediate course, while those from cluster LCS were characterized by the lack or very weak changes in the behavior. In turn, for tonic seizures, two clusters were detected, namely, clusters TS and LTS, including the animals suffering from severe and light tonic seizures, respectively.

**Figure 12:**
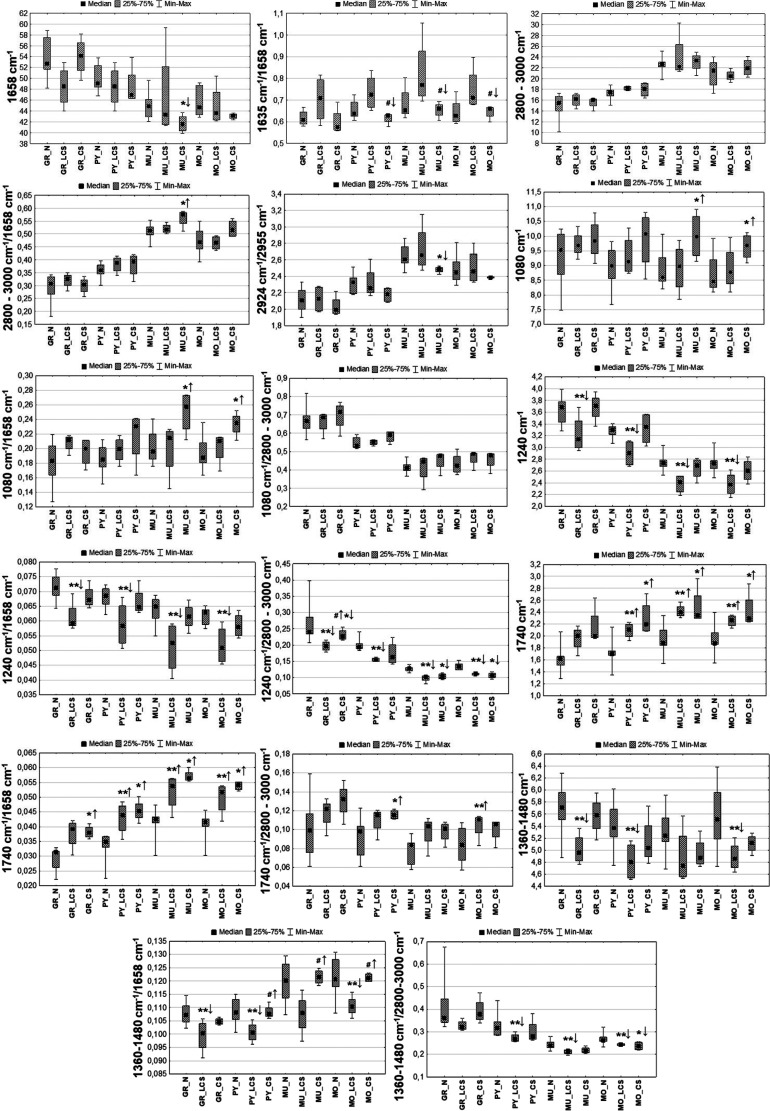
Box-and-whisker plots presenting the spread of the biochemical parameters for animals from CS, LCS, and N groups. Statistically significant differences (Mann–Whitney *U* test, 95% confidence level) between animals representing CS and N groups were marked with *, between LCS and N groups with **, while between the animals presenting the severe and the light clonic seizures with #. Increases and decreases are marked as ↑ and ↓, respectively.

**Figure 13:**
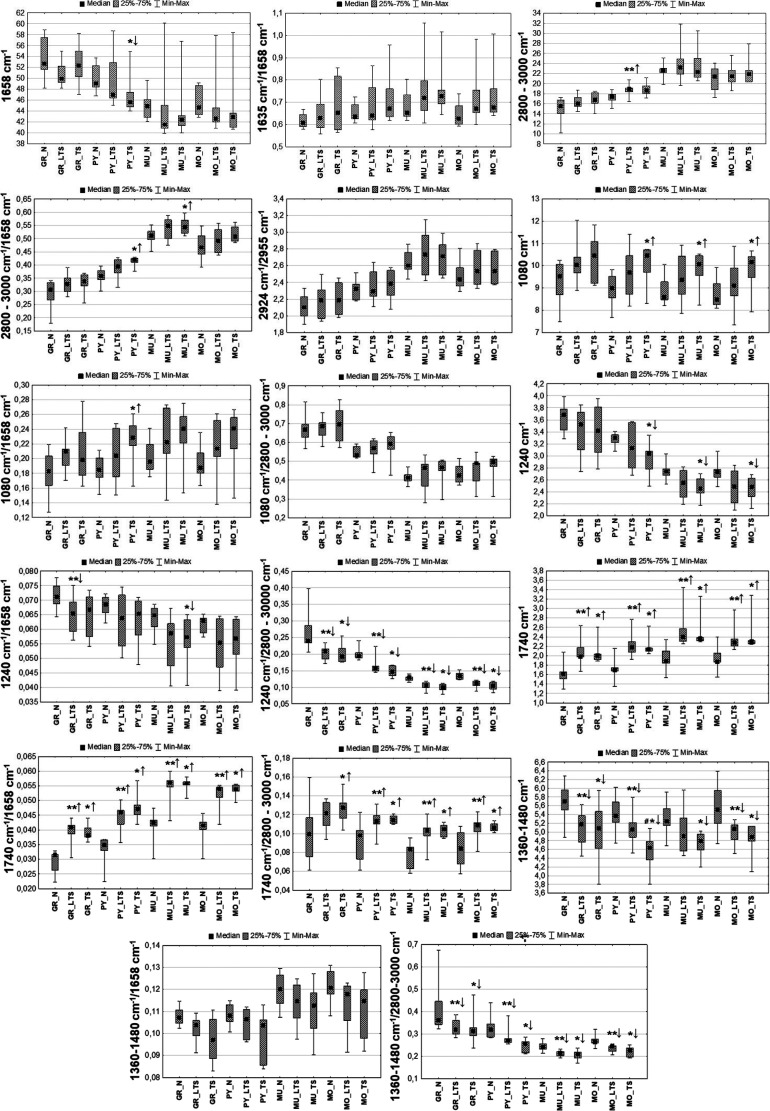
Box-and-whisker plots presenting the spread of the biochemical parameters for animals from TS, LTS, and N groups. Statistically significant differences (Mann–Whitney *U* test, 95% confidence level) between animals representing TS and N groups were marked with *, between LTS and N groups with **, while between the animals presenting the severe and the light TS with #. Increases and decreases are marked as ↑ and ↓, respectively.

To verify if the relationship exists between the biochemical changes appearing within the hippocampal formation and the animal behavior after electrical stimulation, the medians of the examined biochemical parameters were evaluated for subgroups of animals characterized with severe or light tonic and clonic seizures. The data obtained for TS and LTS in the case of tonic seizures and CS and LCS in the case of clonic seizures were compared with the results recorded for normal rats. The statistical significance of the differences between the subgroups of animals subjected to stimulation and controls was tested using the Mann–Whitney *U* test at the confidence level of 95%, and the obtained data are presented in Figures 12 and 13.

As can be seen from Figures 12 and 13, decreased protein accumulation compared to controls was found only in the multiform layer for animals presenting severe clonic seizures (CS group) and in the pyramidal layer in the case of the group of animals characterized with significant tonic activity (TS group). The animals belonging to the CS group showed, moreover, the lower relative level of proteins with the β-type secondary structure compared to rats experiencing light clonic seizures. The mentioned effect was observed for most (except for granular cells) of the examined cellular layers.

The subgroups CS and LCS of animals showing clonic seizures did not present any statistically significant differences in the accumulation of lipids compared to controls. A relevant increase of 2800–3000 cm^–1^ massif intensity in the pyramidal layer was, however, found for rats suffering from light tonic seizures (LTS group). In turn, the relative intensity of the massif with respect to proteins was significantly elevated within the pyramidal layer of the TS group and the multiform layer of CS and TS groups. The opposite relation was found for the intensity ratio of lipid bands at 2924 and 2955 cm^–1^ in the multiform layer in the case of animals showing severe clonic seizures (CS group).

The intensity of the absorption band at 1080 cm^–1^ increased together with the intensification of both clonic and tonic seizures. A statistically significant increase was found for the multiform and molecular layers in the case of the CS group and, additionally, for the pyramidal layer in the case of the TS group. Also, the ratio of the bands at 1080 and 1658 cm^–1^ presented an increasing tendency together with convulsion severity. The mentioned effect was observed for most (except for granular cells) examined cellular layers, but only for some of them, it was statistically relevant.

In animals experiencing light clonic seizures, statistically significant decrease of 1240 cm^–1^ band intensity was found within all cellular layers. In turn, in rats showing severe clonic seizures, the parameter did not differ compared to controls. Exactly the same relations were observed for the ratio of the intensity of the 1240 cm^–1^ and amide I bands. The relative intensity of this band compared to lipids was diminished in all cellular layers and almost always both in animals from the LCS and CS groups. In the case of both light and severe tonic seizures, the absolute and relative intensity of the 1240 cm^–1^ band usually decreased. The changes in the absolute band intensity and its relative content with respect to proteins, however, were not always statistically relevant. In turn, the ratio of the band at 1240 cm^–1^ and lipid massif presented a statistically significant decrease for both light and severe tonic seizures and within all the examined hippocampal layers.

The absolute intensity of the band at 1740 cm^–1^ as well as its relative content with respect to proteins and lipids was elevated in animals presenting both light and severe clonic and tonic seizures. What is more, the vast majority of the differences observed between the animals subjected to electrical stimulation and normal rats was statistically significant.

The absolute and relative intensity of the massif of 1360–1480 cm^–1^ was diminished in rats for which light clonic seizures were observed. The changes in the absolute intensity of the massive and its relative content with respect to proteins (1360–1480 cm^–1^/1658 cm^–1^) and lipids (1360–1480 cm^–1^/2800–3000 cm^–1^) were statistically relevant for most of the examined hippocampal layers. What is more, in the case of the ratio 1360–1480 cm^–1^/1658 cm^–1^, statistically significant differences were observed between the LCS and CS groups for the pyramidal, multiform, and molecular layers.

The animals experiencing both light and severe tonic seizures presented significantly diminished intensity of the massif 1360–1480 cm^–1^, as well as the decrease in its relative intensity compared to the lipid massif 2800–3000 cm^–1^. What is more, in contrast to the clonic seizures, the observed anomalies seem to be independent of behavioral parameters, describing this type of epileptic activity.

## Discussion

TLE is the most common form of epilepsy in adults. What is more, it is characterized by the greatest frequency of drug-resistant cases occurrence, and therefore, there is a great need both to better understand its pathogenesis and look for new strategies of TLE treatment.^1,23^ For this purpose, different animal models of the disease are used, including the kindling model of seizures and/or epilepsy.^24,25^ Such an animal model was also used in our study, and to achieve the kindling phenomenon in rats, repetitive transauricular electroshocks were applied.

To identify the mechanisms of action and examine the effectiveness of the new antiseizure drugs, knowledge on the changes occurring in the brain as a result of seizures in the animal model used for the study is required. Therefore, we determined the influence of repetitive electrical stimulation on the biochemical status of hippocampal formation. The choice of this brain structure for the study was dictated by the fact that it is highly epileptogenic and also susceptible to functional and structural damages appearing in the answer to seizures.^20,26,27^

For the evaluation of biomolecular differences existing in the hippocampal formation between the rats subjected to electrical stimulation and control animals, we used FT-IR microspectroscopy. The analysis of the intensity and distributions of IR bands characteristic for proteins, saturated lipids, cholesterol, and/or its esters, compounds containing carbonyl and phosphate groups showed significant anomalies in both the accumulation and the structure of biomolecules in the examined brain structure after the repetitive electroshocks.

The analysis of the amide I band intensity and the ratio of absorbance at the wavenumbers of 1635 and 1658 cm^–1^ was applied in the work to detect the changes in the protein distribution and structure, respectively. Our study showed a slight decrease in the amide I band intensity in the stimulated animals, but the found relations were not statistically relevant. The increased value of the ratio of the absorbance at 1635 and 1658 cm^–1^ within the molecular layer of rats subjected to electrical kindling may indicate an increased relative level of proteins having the β-sheet secondary structure. Such an effect, according to the existing evidence, may be associated with the progress of oxidative stress and/or the neurodegenerative changes in the nervous tissue.^28−32^ Similar anomalies in the relative content of proteins with the β-type conformation were also found in our previous study on the pilocarpine model of seizures.^16,33^ They usually concerned, however, not only the molecular layer but also other hippocampal layers.

Proteins of the β-sheet structure may cause astrogliosis and accumulation of extracellular glutamate, which contribute to seizure progress.^34^ In turn, the modification of the protein conformation may be induced by the products of lipid peroxidation associated, among others, with oxidative stress promoted by reactive oxygen species.^33,35^ Lipid peroxidation may manifest in some structural changes of these molecules, which can be measured indirectly through the analysis of the ratio of intensities of 2924 and 2955 cm^–1^ absorption bands originating from the asymmetric stretchings of CH_2_ and CH_3_ groups, respectively.^36,37^ Our study, however, did not show any statistically significant anomalies for the mentioned ratio of the bands. On the other hand, the absolute accumulation of lipids and their relative content compared to proteins was elevated within the pyramidal layer of animals subjected to transauricular electroshocks.

The secondary products of lipid peroxidation are aldehydes, and among them, malondialdehyde (MDA) is the most mutagenic and 4-hydroxynonenal (4-HNE) is the most toxic.^38,39^ Both these compounds are used as biomarkers of oxidative damage to lipids occurring during the progress of different diseases.^38,40^ Elevated levels of both MDA and 4-HNE were found in rat brains after 24 h passing from the last seizure in the case of the amygdala kindling model of epilepsy.^41^ In this context, it is necessary to mention that the oxidation of fatty acid side-chains and the fragmentation of peroxides leading to aldehyde production may result in further alterations in cell signaling, DNA damage, and the loss of membrane integrity, followed by the inactivation of their proteins.^38,39^ For animals subjected to electrical stimulation, we observed significantly increased intensity of the 1740 cm^–1^ absorption band originating from the compounds containing carbonyl groups, which may point at the elevated concentration of aldehydes as a result of lipid peroxidation and, the same, seem to confirm the hypothesis concerning the kindling-induced oxidative stress.

The absorption bands occurring at the wavenumbers of 1080 and 1240 cm^–1^ are associated, *inter alia*, with the presence of nucleic acids and phospholipids in the tissue. For this reason, the decrease in their intensity may indicate the damage of DNA and/or alterations in the composition of phospholipids building the cell membranes. Both of these phenomena may follow oxidative stress and may be related to the diseases of the central nervous system (CNS), including epilepsy.^16,31,40^ On the other hand, the absorption band at around 1080 cm^–1^ is also a characteristic for carbohydrates, and therefore, the changes in this spectral region may indicate the differences in the content of these compounds, including fluctuations in glucose levels.^42^ During epileptic seizure progression, increased glucose consumption is observed, which results in a decrease in its level within the regions of the brain with higher convulsive susceptibility.^43^ Therefore, it is rather surprising that the intensity of the 1080 cm^–1^ band was elevated in the hippocampus of experimental animals. However, as their brains were taken around 24 h after the last stimulation, it is probable that we still observed the mobilization of glucose connected with the increased demand for energy during postelectroshock seizures.

The animals subjected to the repetitive electroshocks presented a diminished intensity of the massif of 1360–1480 cm^–1^ in all examined cellular layers. The mentioned spectral region is characteristic for bending vibrations of methyl and methylene groups of lipids, cholesterol, and/or its esters. The quantitative analysis of the lipid content did not show, however, significant changes in their accumulation for most of the examined hippocampal cellular layers in the case of the experimental animals. Therefore, the observed anomalies in the intensity of the 1360–1480 cm^–1^ massif seem to be rather an effect of changes on the content of cholesterol/cholesterol esters than that of lipids.

Cholesterol is of great importance for the proper action of the nervous system, among others, influencing the function of synapses and the release of neurotransmitters.^44−47^ The ability of the CNS neurons for the creation of synapses is restricted by cholesterol availability, and its increased hippocampal efflux may negatively influence the fundamental synaptic physiology, the action of receptors, and both the pre- and postsynaptic plasticity mechanisms.^44,46^ The mentioned anomalies may also impact increased phosphorylation of tau proteins and amyloid accumulation.^44^ The concentration of cholesterol in the CNS depends on the functioning of glial cells producing this sterol and on its delivery through the apoE-containing lipoproteins. The disturbances in the synthesis, transport, or uptake of the cholesterol in the CNS may directly weaken the development of the synaptic circuitry and lead to neurodegenerative diseases.^44,46^ The existing evidence also suggests that cholesterol may have an antioxidant function in normal cell membranes, and its content there may be decreased by the free radicals formed in the damaged tissue.^48,49^ Cholesterol intercepts the oxidants and produces oxysterol compounds, which is a physiological process; however, excess production of these compounds may occur as a result of oxidative stress.^49^ Taking all these into account, the decreased intensity of the massif of 1360–1480 cm^–1^ found in this study may point at the disruption of the cholesterol homeostasis that may be an effect of oxidative stress. However, the relationship between epilepsy and the disorders of cholesterol metabolism is still ambiguous. Increased levels of cholesterol and some oxysterols within the hippocampus were found in a rat model of kainic acid-induced neuronal damage.^50^ In turn, the other study using a mouse model of kainic acid-evoked seizures showed that the levels of cholesterol and 24S hydroxycholesterol (24S-OHC) in this brain area remained unchanged 24 h after SE but was significantly lower compared to controls 48 and 336 h after SE.^51^

Using the cluster analysis, the animals were divided into subgroups differing in the severity of behavioral changes after stimulation. The comparison of the rats showing light and severe clonic seizures with control rats showed that the abnormalities in protein conformation as well as the reduced content of cholesterol and compounds containing phosphate groups, observed after stimulation, are characteristic of animals showing light clonic convulsions. Such relations were not, however, observed in the case of tonic seizures. In turn, the intensity of the band at 1080 cm^–1^ increased with the severity of the seizure activity of both types, which seems to confirm the hypothesis of hippocampal glucose mobilization resulting from increased energy demand.

## Conclusions

The results of the present study confirmed that repetitive stimulation of the brain using transauricular electroshocks leads to significant biomolecular anomalies within the hippocampal formation. The observed abnormalities in the distribution and structure of the examined macromolecules such as increased relative levels of proteins with a β-type secondary structure or an elevated content of compounds containing carbonyl groups may point at the occurrence of processes related to oxidative stress in rats subjected to electrical stimulation. Such a hypothesis seems to be also supported by the found changes in the content of cholesterol and/or cholesterol esters as the disruption of their homeostasis may significantly influence the oxidative stress occurring in cells. The presented results suggest that new antiseizure treatments should be targeted at minimizing the oxidative stress, for example, by including therapeutics, revealing antioxidant activity in the CNS.

## Materials and Methods

### Animals

In the experiment, adult male Wistar rats were used. Animal husbandry, electrical stimulation, seizure intensity assessment, and the preparation of tissue samples were carried out at the Department of Neuroanatomy, Institute of Zoology and Biomedical Research, Jagiellonian University, Krakow. All animal use procedures were approved by the Local Ethical Commission of the Jagiellonian University (agreement no. 40/2010) and were in agreement with the international standards. During the whole life, the rats were maintained under strictly controlled conditions, that is, a constant temperature (20 ± 2 °C) and illumination (12 h light:12 h dark cycle). They obtained solid diet in the form of Labofeed and water *ad libitum*.

Two groups of rats were used for investigation. These were group N containing naive control animals and group K of rats, which from the 60th day of postnatal life were daily, for 21 days, subjected to transauricural electroshocks. The group of electrically stimulated animals consisted of 16 individuals, while the control group included 10 rats. Typically, one sample of the dorsal part of the hippocampus was prepared per brain, and therefore, 26 slices in sum were used for the study.

### Electrical Stimulation and Behavioral Observations

A sinusoidal electric current (*I* = 10 mA, *f* = 60 Hz, *t* = 1 s) was generated by a pair of ear-clip electrodes Rodent Shocker RS type 221 (Hugo Sachs Elektronik—Harvard Apparatus GmbH, Germany). After each stimulation, the animals were observed for 1 h. The secondary tonic and clonic seizures were distinguished and evaluated based on separate scales. The behavioral parameters recorded on a daily basis included the intensity and duration of both types of seizures.

The evaluation of tonic seizures intensity was performed using a scale previously established for seizures induced with electroshock as follows.:^11,52−54^ 0 – no seizures, 1 – forelimb extension without hind limb extension, 2 – complete forelimb extension and partial hind limb extension, and 3 – complete (parallel to the tail) hind limb extension. Clonic seizures were evaluated according to the modified Racine’s limbic seizure scale.^55^ This scale was used in our previous studies on the pilocarpine and electroshock models of seizures.^56^

### Sample Preparation

On the 80th day of life, in the case of normal rats, and after 21 stimulation days, in the case of the K group, the animals were deeply anesthetized with Morbital (Biowet) and perfused with 0.9% saline solution of high analytical quality. The brains were excised and deeply frozen in liquid nitrogen. Twelve micrometer thick slices with the dorsal part of the hippocampal formation,^57^ obtained with a cryomicrotome, were mounted on MirrIR slides and freeze-dried.

### IR Data Collection

The biochemical analysis of rat brain samples was performed using FT-IR microspectroscopy. The measurements were performed at the Faculty of Physics and Applied Computer Science of the AGH University of Science and Technology (Krakow, Poland). A Thermo Scientific Nicolet iN10 MX IR microscope was used for the study. This ultrafast mapping system is equipped with a ceramic radiation source and a linear array of mercury cadmium telluride (MCT) detectors, which was used for the present investigation. The samples deposited on MirrIR slides were analyzed in transflection mode with a spatial resolution of around 25 μm. The spectral resolution was set to 8 cm^–1^, and 32 scans were averaged per spectrum for both the sample and the background. Data acquisition as well as spectral analysis were performed with OMNIC Picta software (version 8.1).

### Spectral Analysis

The IR spectra were collected for the wavenumber range between 900 and 4000 cm^–1^. The analysis of accumulation and distribution of the biomolecules in the hippocampal formation was based on the chemical mapping of their main absorption bands or their ratios. For this purpose, OMNIC Picta, ImageJ (version 1.51j8), and Surfer (version 9.0.) software were used. The characteristics of the examined absorption bands are presented in Table 1.

**Table 1:** Examined Biochemical Parameters^16,31,36,37,58−64^

biochemical parameter	absorption band/ratio of absorption bands
distribution of proteins	1658 cm^–1^ (amide I)
structural changes of proteins (β-sheet to α-helix ratio)	1635 cm^–1^/1658 cm^–1^
distribution of lipids	2800–3000 cm^–1^
saturation level of lipids, changes in the length of fatty acid chains, and the degree of their branching	2924 cm^–1^/2955 cm^–1^
distribution of the compounds containing phosphate groups, including nucleic acids, phospholipids, phosphorylated carbohydrates, differences in the degree of phosphorylation of carbohydrates, and/or glycoproteins	1080 cm^–1^1240 cm^–1^
distribution of lipids, cholesterol esters, and cholesterol	1360–1480 cm^–1^
distribution of phospholipids, cholesterol esters, and ketone bodies	1740 cm^–1^
ratios of appropriate biological compounds	2800–3000 cm^–1^/1658 cm^–1^1080 cm^–1^/1658 cm^–1^1240 cm^–1^/1658 cm^–1^1740 cm^–1^/1658 cm^–1^1360–1480 cm^–1^/1658 cm^–1^1080 cm^–1^/2800–3000 cm^–1^1240 cm^–1^/2800–3000 cm^–1^1740 cm^–1^/2800–3000 cm^–1^1360–1480 cm^–1^/2800–3000 cm^–1^

The two-dimensional chemical maps were generated by imaging the area of one peak or the area ratio of two peaks, including trapezoidal baseline correction. In two cases, we did not examine the intensity of the band, but the integrated absorbance within the particular wavenumber range. These were the regions between the wavenumbers of 2800 and 3000 cm^–1^ and between 1360 and 1480 cm^–1^. For these wavenumber ranges, called massifs in the study, a few absorption bands specific, respectively, to lipids as well as lipids, cholesterol, and cholesterol esters occur in the spectra. The intensity of the massifs was calculated as the integrated absorbance within the mentioned wavenumber ranges. Also, in this case, trapezoidal baseline correction was applied. The details concerning all the taken limits of the bands/massifs and the subtracted background are presented in Table 1S of the Supporting Information.

To quantitatively compare the examined animal groups, the average intensity or the average intensity ratios of the analyzed absorption bands were calculated for four main cellular layers of hippocampal formation, namely, granular (GR), pyramidal (PY), multiform (MU), and molecular (MO) layers.

**Figure 14:**
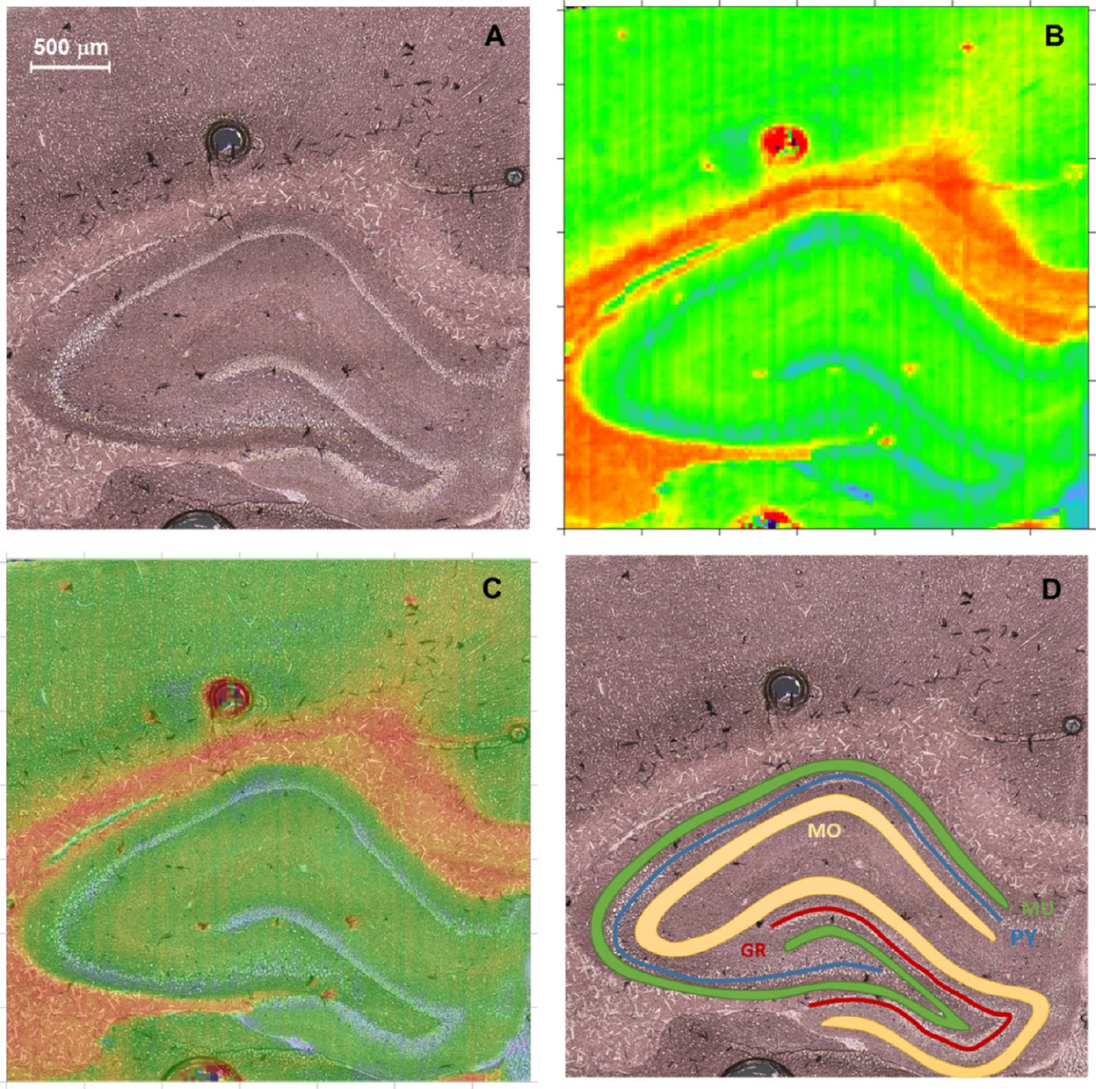
Microscopic view of the selected hippocampal formation (A), the chemical map of the intensity ratio of absorption bands at 1740 and 1658 cm^–1^ (B), and 2D blend of pictures A and B (C). The localization of pixels/areas belonging to the examined cellular layers of hippocampal formation (D). GR, PY, MU, and MO mean granular, pyramidal, multiform, and molecular cell layers, respectively.

To extract the pixels belonging to the particular cellular layer, the chemical maps were imposed on the microscopic pictures of the scanned areas of the brain (see Figure 14C). The quantitative information concerning the intensity of the examined bands or their ratios was taken from pixels belonging to the examined hippocampal areas, avoiding the points localized in the border of the layers (Figure 14D). The mean values of biochemical parameters were calculated independently for each animal and each cellular layer. The number of pixels used in calculations was not lower than 120, 150, and several hundred for granular, pyramidal, and molecular and multiform layers, respectively. The results obtained for the examined rat groups/subgroups were then compared, and the observed differences were evaluated using appropriate statistical tools.

### Statistical Analysis

The Mann–Whitney *U* test was applied for the statistical evaluation of the differences between N and K groups, as well as the subgroups of electrically stimulated animals. The choice of the nonparametric statistical test was dictated by the fact that our data could not meet the assumptions about normality, homoscedasticity, and linearity, which are necessary for the use of its parametric alternative. In the present study, the statistical significance of the observed differences was examined at the significance level of 5% (*p*-value < 0.05), and the statistical analysis was performed with the STATISTICA software (version 7.1).

## Supporting Information Available

Details of the chemical mapping process. Variation of chemical maps obtained for normal rats and animals subjected to the repetitive electrical stimulation (PDF)
