# Supplementary material for: The Use of Fourier Transform Infrared Microspectroscopy for the Determination of Biochemical Anomalies of the Hippocampal Formation Characteristic for the Kindling Model of Seizures
Source: ACS Chem Neurosci. 2021 Nov 24;12(24):4564–79. doi: 10.1021/acschemneuro.1c00642 (PMC8678993; doi:10.1021/acschemneuro.1c00642)
Supplement: Supplementary file 1 — cn1c00642_si_001.pdf [file cn1c00642_si_001.pdf]

Supplementary materials

Table 1S. The details of chemical mapping process.

Absorption band/ ratio of absorption bands [cm^{-1}]	Range for the numerator [cm^{-1}]	Baseline for the numerator [cm^{-1}]	Range for the denominator [cm^{-1}]	Baseline for the denominator [cm^{-1}]
1658	~1600-1715	~1480-1770	-	-
1635/1658	~1635	~1480-1770	~1658	~1480-1770
2800-3000	~2800-3000	~2800-3000	-	-
2800-3000/1658	~2800-3000	~2800-3000	~1600-1715	~1480-1770
2924/2955	~2880-2945	~2800-3000	~2945-3000	~2800-3000
1080	~1000-1140	~1000-1140	-	-
1080/1658	~1000-1140	~1000-1140	~1600-1715	~1480-1770
1080/2800-3000	~1000-1140	~1000-1140	~2800-3000	~2800-3000
1240	~1190-1280	~1190-1280	-	-
1240/1658	~1190-1280	~1190-1280	~1600-1715	~1480-1770
1240/2800-3000	~1190-1280	~1190-1280	~2800-3000	~2800-3000
1740	~1720-1770	~1480-1770	-	-
1740/1658	~1720-1770	~1480-1770	~1600-1715	~1480-1770
1740/2800-3000	~1720-1770	~1480-1770	~2800-3000	~2800-3000
1360-1480	~1360-1480	~1360-1480	-	-
1360-1480/1658	~1360-1480	~1360-1480	~1600-1715	~1480-1770
1360-1480/2800-3000	~1360-1480	~1360-1480	~2800-3000	~2800-3000

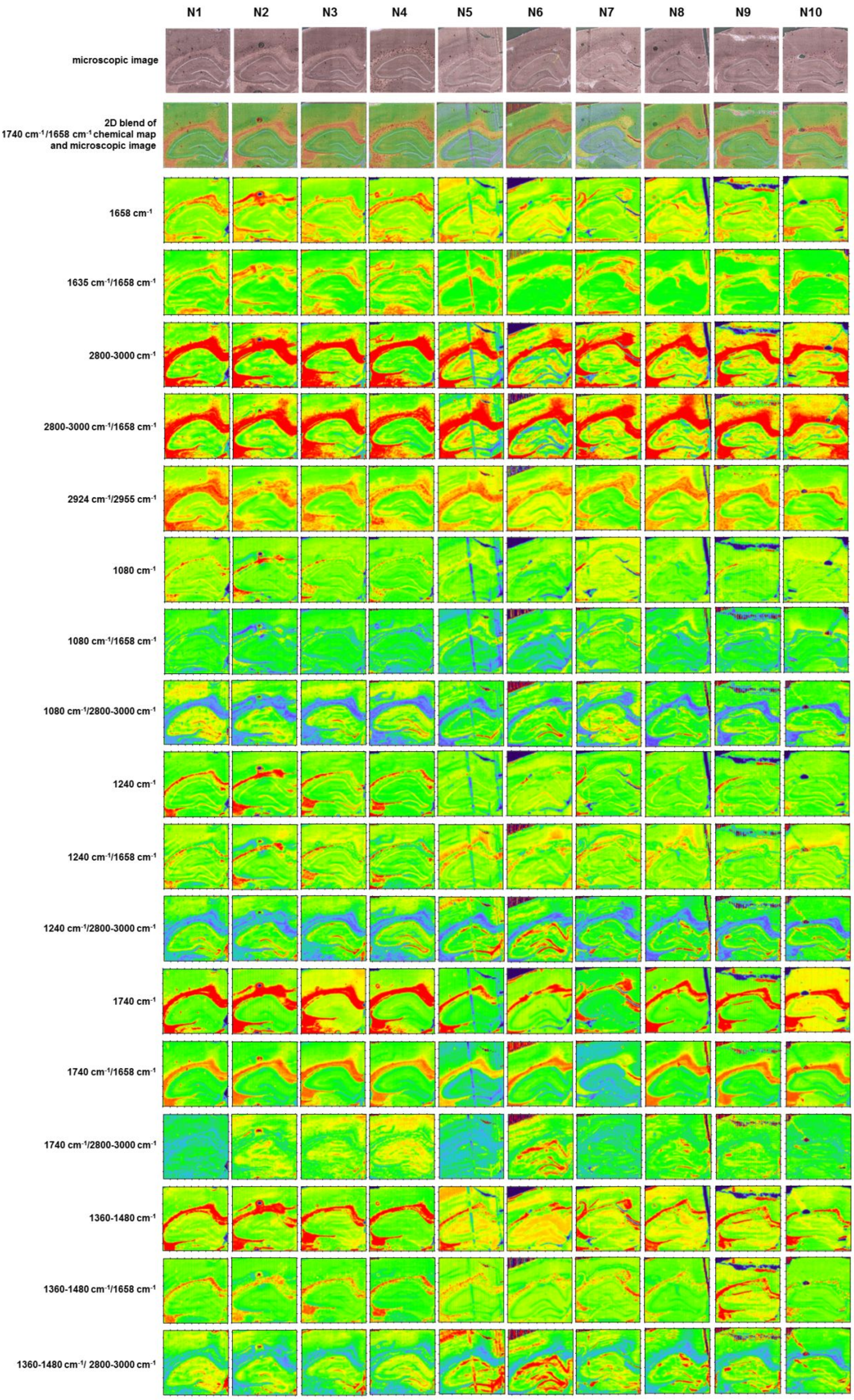

Figure 1S. The comparison of chemical maps obtained for 10 normal rats.

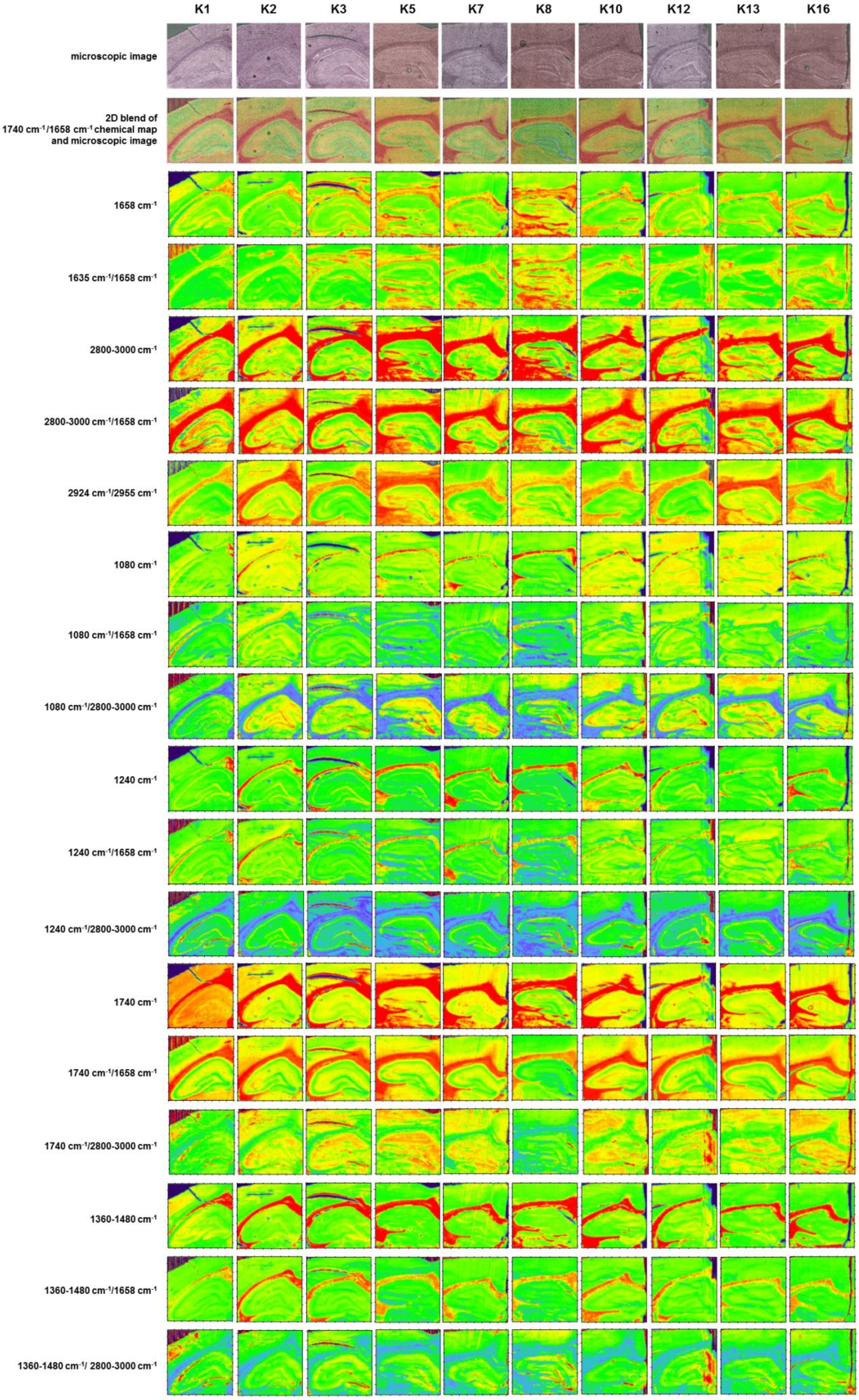

Figure 2S. The comparison of chemical maps obtained for 10 randomly selected animals subjected to the repetitive electrical stimulation.
